# Is a Decentralised Health Policy Associated With Better Self-rated Health and Health Services Evaluation? A Comparative Study of European Countries

**DOI:** 10.34172/ijhpm.2020.13

**Published:** 2020-02-22

**Authors:** Pål E. Martinussen, Håvard T. Rydland

**Affiliations:** Department of Sociology and Political Science, Norwegian University of Science and Technology (NTNU), Trondheim, Norway.

**Keywords:** Decentralisation, Self-rated Health, Health Services Satisfaction, Europe, Multi-level Methods

## Abstract

**Background:** While decentralisation has come to be a major policy strategy in many healthcare systems, there is still insufficient evidence about its impact. Most studies have been of developing countries, and they have provided mixed results. This study is the first to test the relevance of political decentralisation across European countries, thus meeting the demand for more studies of decentralisation in developed countries, and building on an indicator of decentralisation reflecting the allocation of authority for both health policy tasks and health policy areas.

**Methods:** As indicators of health system outcome, we employed 2 measures that have not previously been investigated in the context of decentralisation: self-rated health and satisfaction with healthcare system. Using multilevel modelling and controlling for individual-level demographic and socioeconomic variables, the paper utilised the 2014 (7th) and 2016 (8th) round of the European Social Survey (ESS), including more than 70 000 individuals from 20 countries.

**Results:** The results suggest that decentralisation has a positive and significant association with health system satisfaction, but not with self-rated health. Of the different operationalisations, decentralised healthcare provision had the strongest association with health system satisfaction.

**Conclusions:** Our study fails to provide clear support for decentralised health systems. There is a need for more research on the impact of such reforms in order to provide policy-makers with knowledge of desirable governance, organisational designs, management and incentives in healthcare.

## Background

Decentralisation has become the buzzword of health policy over the last decades. The term decentralisation refers to a wide variety of power transfer arrangements and accountability systems, but generally builds on the idea that smaller organisations are more responsive and accountable than larger organisations. Decentralisation is thus seen as a response to poor efficiency, slow innovation, and lack of responsiveness to patient’s demands, which may be some of the drawbacks of large, centralised public institutions. If properly designed and implemented, decentralisation is expected to improve equity, efficiency, quality, and access to healthcare services and ultimately health outcomes.^1^ However, while decentralisation has come to be a major policy strategy in many healthcare systems,^2-4^ there is still insufficient evidence about its impact. Most studies of the impacts of decentralisation have been of low- and middle-income countries (LMICs), and they have provided mixed results. A recent review of the literature concluded that there is no rigorous evidence of the impact of decentralisation on health system performance or outcomes.^5^ Another systematic review of decentralisation of health systems in LMICs found that decentralisation of governance, financing and service delivery had positive effects on the system, while the decentralisation of resource management has proven more challenging.^6^

This study investigates the role of decentralisation for health system performance and outcome concentrating on European countries, thus meeting the demand for more studies of decentralisation in developed countries. Building on a measure of the allocation of authority in European health policy proposed by Adolph and colleagues,^7^ we analyse whether degree of decentralisation is associated with self-rated health and evaluation of health services, while controlling for important individual and country-level determinants, and using the most recent data on Europe available.

The literature on health system decentralisation generally distinguishes between 3 outcome domains: health system actions and inputs (eg, health system budgets and spending); health system performance outcomes (eg, health insurance coverage, availability of appropriate health services/supplies); and health outcomes (eg, maternal or child health indicators).^5^ Our study addresses the 2 latter outcome domains: performance and health outcomes. The indicators employed within the *health system performance outcomes* domain typically reflect the effects on governance, access to medicines and equipment, health information systems, human resources, and service delivery.^6^ In this study, we take a different approach, using instead individual evaluations of a country’s health services to reflect health system performance. Public satisfaction with health services is important for several reasons. Citizens are beneficiaries and actors in health systems, and their opinions can be important in shaping health policies. They can provide feedback on the quality and responsiveness of services, and may bring legitimacy and accountability to the policy-making process.^8-10^ Measures on satisfaction are increasingly used in international comparisons of health systems. Healthcare satisfaction is an alternative way to measure healthcare quality and performance, and the results are typically used to identify whether a system is performing sufficiently well and to identify areas where it can improve.^11^ There has consequently been growing interest in measuring satisfaction with the health services and health system performance.^11-13^ Public satisfaction has therefore emerged as a common measure of health system performance in the literature.^13-19^

The studies on the effects of decentralisation on *health outcomes* have mainly focused on infant mortality rate and post neonatal mortality,^6^ thus excluding other relevant indicators of health outcomes. This study focuses on self-rated health, which is untested in the context of healthcare decentralisation. Self-rated health has proved to be a consistently reliable predictor of mortality, and even though variation between population subgroups has been documented, self-rated health often exceeds the reliability of more objective measures.^19^ Self-rated health is affected by several factors, among those vitality, physical function, bodily pain, mental health, role physical, social functioning.^20^ Looking at the reliability of self-reported morbidity, Dalstra and colleagues^21^ found, in a review of socioeconomic differences in chronic conditions, a high degree of accuracy between self-reported conditions and physician-reported medical histories. The few diverging incidents were individuals of low socioeconomic status who underreported certain conditions, with the consequences being that researchers potentially underestimate socioeconomic health inequalities. Also, in a landmark meta-analysis of 27 community studies, Idler and Benyamini^22^ found self-rated health to be a significant, independent predictor of mortality in nearly all studies, thereby proving the measure’s applicability across different contexts. A large body of literature concerning self-rated health, its determinants, and its outcomes has accumulated from studies conducted throughout the world,^23^ and self-rated health is now commonly used as an indicator of health status in welfare and healthcare studies,^24-26^ as well as in other research fields.^27,28^

We used multilevel logistic and ordinary least squares regression analysis on the seventh and eighth rounds of the European Social Survey (ESS) from 2014 and 2016, including approximately 70 000 respondents from 20 countries. The individual-level variables in the analysis included age, gender, economic situation and education. Preliminary intra-class correlation analyses led us to believe that some explanations of variance in the dependent variable were to be found at the country level. We further controlled for healthcare spending at the country level, and a health outcome indicator at the regional level.

The Argument for Decentralisation

The argument for decentralisation originates from the traditional theory of fiscal federalism. The main assumption is that by allowing a close match between the provision of ‘local’ public goods and services and citizen’s wishes, and a greater experimentation and innovation in the production of these goods and services, both allocative and productive efficiency will increase.^29^ The beneficial impact of decentralisation of health services is based on the assumption that it can improve the information of local decision-makers about local circumstances, stimulating prompt and effective responses to local needs, and that it may serve as an effective channel for people to express their preferences. Furthermore, local decision-makers have more opportunities to reduce costs than central managers: they can tailor staff and procedures to the local context, and have more freedom to experiment with alternative ways of doing things and to implement them rather than relying on centrally determined procedures. Decentralisation is thus expected to improve health system performance and outcomes through higher equity, efficiency, quality, and access to healthcare services.^1^

While there are compelling theoretical arguments for devolution of policy-making in health services, decentralisation is however not without its limitations.^30-31^ The arguments put forward for centralisation are mainly economic, and are relevant for healthcare. First, central intervention may be considered necessary to prevent inefficient location of facilities (such as hospitals) by local decision-makers accountable to local electors. Furthermore, centralised healthcare may lead to more efficient pricing of inputs by a single purchaser of healthcare. Centralisation can also prevent local authorities, under pressures to raise their own revenues, from relying on user fees to finance their services or to reduce the coverage of the universal health package. Hence, unless central government coordinates an adequate transfer mechanism from richer to poorer regions, decentralisation may result in increased inequalities in healthcare. Finally, centralisation of health services with important externalities, such as immunisation services, could encourage local decision-makers to “free-ride” on the immunisation status of their neighbours, which could lead to a sub optimal disease protection level provided in the country as a whole.^1,32^

A review of the decentralisation efforts in Europe shows that they consist of a large variety of political, economic, organisational, and legal variants of decentralisation, each supported by its own specific logic.^33^ The health-related powers of these decentralised bodies ranges from nearly independent decision-making to serving as little more than administrative paper-processors for the national government, and the decentralised bodies themselves may be publicly operated institutions (tax-funded countries), not-for-profit private bodies (sickness funds in social health insurance countries), or profit-making companies listed on the stock exchange. Although decentralisation has been widely implemented in health systems, there is consequently little agreement about how to define it or the outcomes it should produce.

Evidence on the Effect of Decentralisation in Healthcare

The literature on the impact of decentralisation in healthcare has almost exclusively dealt with LMIC. Two recent systematic reviews of the research field show that previous studies have investigated a great variety of quantitative indicators on the effects of decentralisation, such as governance, financing, access to medicines and equipment, health information systems, human resources, service delivery, utilisation of health services, infant or post-neonatal mortality and health insurance coverage.^5,6^ Given that our focus in this study is on health system performance and outcomes, we consequently limit our literature review to those 2 dimensions.

The literature on *health outcomes* has mainly measured decentralisation in fiscal terms, as the proportion of total expenditure or revenue accounted for by sub-national governments, and using infant mortality rate as the outcome measure.^5^ In general, the evidence indicates a beneficial impact of fiscal decentralisation on population health.^1,32^ Habibi et al^34^ studied 23 Argentinian provinces over a 25-year period (1970-1994), and the results suggested that fiscal decentralisation reduced intraregional disparities and increased levels of human development (ratio of students enrolled in secondary school per one thousand primary students and infant mortality rate). Using a panel data set of 29 Chinese provinces from 1980 to 1993, Yee^35^ concluded that fiscal decentralisation had been beneficial to mortality rates (and local expenditure on health). Also building on data from Chinese provinces, Jin and Sun^36^ used a panel data set for the period 1980-2003 to investigate the impact of fiscal decentralisation on infant mortality rate. Controlling for number of medical beds and doctors per 10 000 persons, they found that, contradictory to the predictions made by the conventional theories, fiscal decentralisation had generated an overall adverse impact on the infant mortality rate in China. Furthermore, Asfaw et al^37^ studied the impact of fiscal decentralisation on rural infant mortality rates in India between 1990 and 1997. Also controlling for real per capita income and percentage of literate women, the results showed that fiscal decentralisation played a statistically significant role in reducing rural infant mortality rate and that the effectiveness of fiscal decentralisation can be affected by other complementary factors such as the level of political decentralisation. A recent contribution from Samadi et al^38^ that used panel data from Iran between 2007 and 2010 found that fiscal decentralisation in the health sector had a negative impact on under-five mortality and fiscal decentralisation in provincial revenues a positive impact. The study also controlled for physician density, hospital bed density, Gini coefficient, unemployment rate and urbanisation.

In a rare study of a Western country, Cantanero and Pascual^39^ used a panel of Spanish regions for the period 1992-2003 to explore the impact of fiscal decentralisation on infant mortality rates and life expectancy. Their model also included real per capita income, acute care beds (density per 1000 population) and general practitioners (density per 1000 population), and the results suggested that fiscal decentralisation was associated with lower infant mortality rates and greater life expectancy. In another study, Rubio and colleagues^32^ used panel data of the highly decentralised Canadian provinces during the period 1979 to 1995 to investigate the impact of fiscal decentralisation on infant mortality. Health decentralisation was defined as the proportion of sub-national health spending over total health expenditure, and the analysis included control variables to account for social capital (education), needs (low birth weight) and maternal tobacco consumption. The results indicated that fiscal decentralisation had a positive and substantial influence on the effectiveness of public policy in improving a population’s health over the period studied.

Few studies have included cross-country analyses of the relationship between decentralisation and health outcomes. A rare exception is Robalino et al,^40^ who used a panel of LMICs covering the period 1970-1995 to study the effect of fiscal decentralisation on infant mortality rates, while also controlling for gross domestic product per capita, ethno-linguistic fractionalisation, corruption and political rights. The study found that higher fiscal decentralisation was consistently associated with lower mortality rates, and that benefits of fiscal decentralisation are particularly important for poor countries. A study by Rubio et al^1^ introduced an improved measure of fiscal decentralisation that grouped taxes according to the level of discretion entitled to local governments, thus reflecting better the existence of autonomy in the decision-making authority of lower tiers of government, which is a crucial issue in the decentralisation process. Also, contrary to much of the previous literature, the study investigated the impact of decentralisation on health outcomes in developed countries by using a panel of 19 Organisation for Economic Co-operation and Development (OECD) countries from 1965 to 2001. Health outcome was measured through infant mortality, and control variables included the level of medical care inputs (number of doctors per 1000 population), gross domestic product per capita, educational level and consumption of alcohol and tobacco. The results showed that fiscal decentralisation had a substantial and positive effect on health outcomes over the period studied, and that conventional measures of decentralisation tend to over-estimate the magnitude of the effect.

The studies addressing *health system performance outcomes* have employed a far more varied selection of decentralisation indicators, also covering aspects such as the timing of decentralisation,^41,42^ the adoption of a decentralisation framework^43,44^ and the degree of mobilised health resources^45,46^ in addition to the usual fiscal autonomy constructs. A study based on repeated cross-sectional individual data from Tanzania investigated the timing of decentralisation on utilisation of skilled birth attendants, and found a harmful effect among wealthy population groups and a beneficial effect among the poor population.^42^ In a similar study based on Indonesian data, Hodge et al^41^ also documented both beneficial and harmful effects: the probability of delivering birth at a health facility increased after the implementation of decentralisation, while regional disparity increased. Faguet and Sánchez^47^ used longitudinal data from Colombian municipalities in the period 1993-2004 to document a positive impact of fiscal decentralisation on change in the poor population covered by public health insurance. Another study examined the impact of decentralisation on childhood immunisation, using a time-series data set of 140 LIMCs in the period 1980-1997.^44^ The study documented different effects of decentralisation in LMICs: decentralised low-income countries had higher coverage rates than centralised low-income countries, while the reverse effect was observed for the middle-income countries. Also investigating immunisation status among children, Maharani and Tampubolon^46^ found no effect of fiscal decentralisation in a study based on cross-sectional individual data from Indonesia. A further study based on data from Indonesia found that fiscal decentralisation was associated with higher outpatient healthcare utilisation rates.^45^

Several controversies and gaps thus remain in the study of the impacts of decentralisation on health system performance and outcomes. First, a large majority of the literature has focused on developing countries. Given how decentralisation has emerged as a major health policy also in Europe, it is surprising how few studies that have evaluated decentralisation in European countries.

Secondly, most studies have used panel data from provinces from a single country as the unit of analysis. This approach has typically involved province-level fixed effects models or general cross-sectional linear regression. Only a few studies^1,40^ have employed cross-country comparisons, or focused on developed countries.^1^ No previous studies have utilised a multilevel design, enabling controls for how both important individual- and country-level characteristics influence health system outcomes.

Third, the favoured health outcome indicators have been mortality rates, infant mortality, low birth weight and life expectancy, while the performance measures have included fiscal autonomy constructs and various attempts to reflect the timing, adoption and degree of decentralisation. As argued above, the concepts of self-rated health and health service evaluation have several advantages, and studies employing these as indicators of health system performance and outcome should therefore supplement the studies that have used the more common indicators.

Our study adds to the research field by attempting to fill some of these gaps. To date, no studies have used multilevel analysis to examine the relationship between decentralisation and health system performance measured by self-perception of health status and patient satisfaction. In the following we outline a multilevel model to investigate how decentralisation is associated with self-rated health and health services evaluation, while controlling for important individual and country-level determinants, and using the most recent data on Europe available.

## Methods

We used the seventh and eighth wave of the ESS as our main data source. Approximately 70 000 respondents from 20 countries and 44 country-year groups were included in our final models.^48^ Response rates varied from ~30% to ~70%, overall similar to previous rounds of the survey (for more information particularly on ESS7, see Eikemo et al^49^). Country level data was collected from OECD health statistics^50-52^ and the Health in Transition (HiT) series from the European Observatory on Health Systems and Policies.^53^ The ESS datafile also included regional level data collected from Eurostat. Data is weighted using a combined design and sample weight, adjusting for both the probability of being included because of sampling design and the number of respondents from each country. In the following, dependent and independent variables are presented; descriptive statistics of these can be found in Tables 1 and 2.

**Table 1:** Descriptive Statistics, Country Level

	**Provision**	**Implement**	**Finance**	**Public Health**	**Secondary/Tertiary Care**	**Primary Care**	**Index**	**PHE**
Austria	3	3	0	2	2	2	1	3.76
Belgium	3	3	0	2	2	2	1	3.59
Czech Republic	1	1	0	0	1	0	1	2.01
Denmark	3	3	3	3	3	3	2	4.02
Estonia	2	0	0	1	1	0	1	1.34
Finland	3	3	3	3	3	3	2	2.96
France	3	0	0	1	1	1	1	3.49
Germany	2	3	0	2	2	1	1	4.33
Hungary	2	0	0	0	1	1	1	1.23
Ireland	0	0	0	0	0	0	0	3.61
Italy	3	3	3	3	3	3	2	2.51
Lithuania	3	0	0	1	1	1	1	1.17
The Netherlands	1	1	1	3	0	0	1	4.29
Poland	2	0	0	1	0	1	1	1.15
Portugal	0	0	0	0	0	0	0	1.73
Slovenia	0	0	0	0	0	0	0	1.90
Spain	3	3	3	3	3	3	2	2.18
Sweden	3	3	3	3	3	3	2	4.31
Switzerland	2	2	0	0	2	2	1	4.61
United Kingdom	3	3	3	3	3	3	2	3.16

Abbreviation: PHE, public health expenditure in US$1000 per capita.

**Table 2:** Descriptive Statistics, Individual Level

**Variable**	**Distribution/Mean**	**SD**	**Min-Max**
Self-rated health (good)	92.3%	0.90	-
Satisfaction with health system	5.63	2.47	0-10
Age	49.7	18.5	14-112
Gender (woman)	52.9%	-	-
Primary education	27.6%		
Secondary education	50.1%	-	-
Tertiary education	22.4%		
Financial strain	20.5%	-	-
N	71 868	-	-

### Dependent Variables

We employed 2 dependent variables in the analyses. First, we used a variable where respondents were asked to report *the general state of their health*, using a scale from 1 to 5. The variable is slightly skewed, since a majority of the respondents have responded 3, 4 or 5, and was therefore dichotomised, with responses ‘bad’ and ‘very bad’ coded as 0 and responses ‘fair,’ ‘good’ and ‘very good’ coded as 1. Although the use of the dichotomous version and the original 5-point variable returns quite similar results, tests show that the assumption of homoscedasticity is violated with the 5-point variable, which means that a logistic approach should be employed.

The second variable builds on a question where respondents were asked what they think overall about *the state of health services* in their home country nowadays, using a scale from 0 (‘extremely bad’) to 10 (‘extremely good’). Answer categories ‘refusal,’ ‘don’t know,’ and ‘no answer’ were coded as items missing; the variable was otherwise kept as is.

### Main Explanatory Variable: Decentralisation

We based our measure of decentralisation on the work of Adolph et al.^7^ They used the peer-reviewed HiT series of country profiles of the European Observatory on Health Systems and Policies^ [1]^ to classify the allocation of authority for health policies in 28 European countries. This measure classifies policies along 2 variables: the first variable divides health policies into 5 tasks (framework legislation, implementation legislation, finance, provision and regulation), while the second variable designates 4 health policy areas (pharmaceuticals, primary care, secondary-tertiary care, and public health). The indicator coded the primary allocation of authority – to the central state, the region, or localities – for each of the 16 policy task-area combinations. We employed their classification of the policy tasks provision, implementation, and finance in the policy areas public health, secondary/tertiary healthcare, and primary healthcare. Countries were given the score of 2 on the decentralisation index if authority was located at the local or regional level for all 3 policy tasks and all 3 policy areas. First, we constructed 6 task- and area-specific variables, all ranging from 1 to 3; for example, a country was given the score 3 on the decentralised healthcare provision variable if the provision of public health, secondary/tertiary healthcare, and primary healthcare all were decentralised. Second, we constructed an overall decentralisation index where countries were given the score of 0 if authority of all tasks and areas was located on the state level. Intermediate countries with a mix of centralised and decentralised health policy authority were given the score of 1. Some of our study countries were not included in Adolph and colleagues’ article: in these cases we assessed the relevant HiT profiles. An overview of the country-level variables is displayed in Table 1.

### Control Variables, Individual Level

Variables measuring individual features function as control variables, and have the following characteristics. *Age* is a numerical variable, which was entered into the models as a mean centred and a squared term. This was done to account for curve-linearity and remove outliers with ages below 20 or above 79. *Gender* is a dichotomous variable, where women were given the value 1. Two measures of socio-economic background were included. *Highest completed education level* (primary, secondary, or tertiary, with the latter as reference category) was measured using the International Standard Classification of Education. In addition, *financial strain* measured whether respondents are coping or living comfortably on present income (coded 0), or whether they find it ‘difficult’ or ‘very difficult’ (coded 1)^ [2] ^.

### Control Variables, Regional Level

Since decentralisation is a concept that is fundamentally related to health at the regional level, a regional measure of *life expectancy* was included. This is the mean number of years a person in each region is expected to live at age less than one year. The regional level is the highest level below the ‘national’ level, NUTS 1 (nomenclature d’unités territoriales statistiques – an EU standard for administrative diversions within countries) where available. The variable was gathered from Eurostat and prepared by ESS.

### Control Variables, Country Level

At country level we controlled for *healthcare financing*, which was measured as public health expenditure in US$1000 per capita (the natural logarithm was used in the model). This variablereflects the interventionist power of the state in the field of healthcare.^18^ The data was gathered from OECD Health Financing Statistics.^50^ An emphasis on public spending is expected to influence self-rated health and overall healthcare satisfaction positively. Obviously, there are a number of other country-level factors that could potentially affect the relationship between decentralisation and satisfaction/self-rated health. A rule of thumb in multilevel modelling suggests 10 observations per included variable, and given that the number of countries in our data was limited to 20, we did not add any additional country-level control variables. However, in sensitivity analyses (see Supplementary file 1, Tables S1-S8) we included additional country-level control variables reflecting out-of-pocket spending on healthcare (as a percentage of total health expenditure), healthcare provision (measured as general practitioners per 1000 inhabitants), and access regulation (an index adopted from Wendt et al^54^). Finally, we also added a survey wave dummy-variable to the analysis, in order to control for shared trends on the dependent variables.

### Multilevel Modelling

We used multilevel regression analysis with the presumption that our cross-country data is nested: individual respondents in regions, which are further nested in countries. This means that variables and residuals are located at 3 levels: individual, regional, and country-level. For models with self-rated health as the dependent variable, we used a logistic regression model; for models with health system satisfaction, we used standard linear mixed-effects models. We first estimated an empty ‘baseline’ model without independent variables, in order to calculate the intra-class correlation of the dependent variable. Approximately 5% of the total variance in self-rated health is attributable to the country-level, and the corresponding figure for health system satisfaction is around 21%. The corresponding figures for region-level variance were 1% and 3%. Next, we added the individual-, regional-, and country-level variables, resulting in a total of 7 models in addition to the baseline: decentralised provision, implementation, financing, public health, secondary/tertiary healthcare, primary healthcare, and finally the decentralisation index.

In addition, we performed several sensitivity tests, including models using the original 5-point self-rated health variable, and expanding models stepwise with the regional and country level control variables. We also estimated our models with fixed effects for regions in order to control for regional variation that was not picked up by the region-level life expectancy variable. As this did not change our results, we have chosen not to present these results here.

## Results

The results indicate that no operationalisations of decentralisation had significant associations with respondents’ self-rated health (Tables 3 and 4). Sensitivity analyses controlling for out-of-pocket spending, healthcare provision, and access regulation returned similar results, as did models using linear multilevel regression on the original 5-point self-rated health variable. However, several indicators of decentralisation showed significant, positive associations with health system satisfaction. Decentralised provision of healthcare was associated with a 0.4 increase in health system satisfaction. Decentralisation of a task related to secondary or tertiary healthcare was associated with a 0.3 increase in satisfaction. Finally, intermediate or high levels of overall healthcare decentralisation, measured by the index, was associated with increased satisfaction by respectively 1.3 and 1.1 points. Additionally, in our sensitivity analyses, also decentralised healthcare implementation, public healthcare, and primary healthcare returned significant associations with healthcare satisfaction.

**Table 3:** Regression Results, Self-rated Health (Logits)

	**Model 1**	**Model 2**	**Model 3**	**Model 4**	**Model 5**	**Model 6**	**Model 7**
Age (squared)	0.000 194^***^ (0.000 055 8)	0.000 194^***^ (0.000 055 8)	0.000 193^***^ (0.000 055 8)	0.000 193^***^ (0.000 055 8)	0.000 194^***^ (0.000 055 8)	0.000 193^***^ (0.000 055 8)	0.000 194^***^ (0.000 055 8)
Age (centered)	-0.0673^***^ (0.005 27)	-0.0673^***^ (0.005 27)	-0.0672^***^ (0.005 26)	-0.0673^***^ (0.005 26)	-0.0673^***^ (0.005 27)	-0.0672^***^ (0.005 27)	-0.0673^***^ (0.005 26)
Gender (woman)	-0.166^**^ (0.0541)	-0.166^**^ (0.0541)	-0.166^**^ (0.0541)	-0.166^**^ (0.0541)	-0.166^**^ (0.0541)	-0.166^**^ (0.0541)	-0.166^**^ (0.0541)
Primary education	-0.829^***^ (0.0655)	-0.829^***^ (0.0656)	-0.828^***^ (0.0655)	-0.828^***^ (0.0658)	-0.829^***^ (0.0657)	-0.829^***^ (0.0655)	-0.829^***^ (0.0657)
Secondary education	-0.450^***^ (0.0639)	-0.450^***^ (0.0639)	-0.450^***^ (0.0640)	-0.451^***^ (0.0640)	-0.450^***^ (0.0639)	-0.450^***^ (0.0639)	-0.450^***^ (0.0639)
Financial strain	-1.145^***^ (0.0693)	-1.145^***^ (0.0694)	-1.145^***^ (0.0693)	-1.145^***^ (0.0694)	-1.145^***^ (0.0694)	-1.145^***^ (0.0693)	-1.145^***^ (0.0694)
Survey wave dummy (2016)	-0.0911 (0.0530)	-0.0970 (0.0541)	-0.0915 (0.0523)	-0.0961 (0.0534)	-0.0937 (0.0530)	-0.0905 (0.0532)	-0.0918 (0.0526)
Life expectancy (region)	0.0383 (0.0342)	0.0412 (0.0325)	0.0418 (0.0323)	0.0414 (0.0323)	0.0408 (0.0327)	0.0398 (0.0332)	0.0399 (0.0331)
PHE (ln)	0.370 (0.259)	0.454 (0.303)	0.365 (0.261)	0.443 (0.265)	0.404 (0.265)	0.354 (0.278)	0.376 (0.258)
Decentralisation provision	-0.0475 (0.0847)						
Decentralisation implementation		-0.0631 (0.0937)					
Decentralisation financing			-0.0320 (0.0689)				
Decentralisation public healthcare				-0.0921 (0.0740)			
Decentralisation secondary/tertiary care					-0.0614 (0.0815)		
Decentralisation primary care						-0.008 32 (0.0820)	
Decentralisation index = 1							-0.168 (0.286)
Decentralisation index = 2							-0.217 (0.306)
Constant	-2.468 (1.631)	-3.362 (2.096)	-2.780 (1.799)	-3.245 (1.705)	-2.939 (1.784)	-2.554 (1.807)	-2.637 (1.620)
Country-level variance	0.122^**^ (0.0438)	0.122^**^ (0.0410)	0.123^**^ (0.0471)	0.116^**^ (0.0423)	0.121^**^ (0.0431)	0.124^**^ (0.0463)	0.121^**^ (0.0446)
Region-level variance	0.0382^**^ (0.0129)	0.0383^**^ (0.0129)	0.0383^**^ (0.0128)	0.0382^**^ (0.0129)	0.0383^**^ (0.0129)	0.0383^**^ (0.0129)	0.0383^**^ (0.0129)
N	71 868	71 868	71 868	71 868	71 868	71 868	71 868

Abbreviation: PHE, public health expenditure in US$1000 per capita. Standard errors in parentheses. ^+^ *P *< .1 ^*^ *P *< .05, ^**^ *P *< .01, ^***^ *P *< .001.

**Table 4:** Regression Results, Health System Satisfaction

	**Model 1**	**Model 2**	**Model 3**	**Model 4**	**Model 5**	**Model 6**	**Model 7**
Age (squared)	0.000 752^***^ (0.000 0627)	0.000 752^***^ (0.000 062 7)	0.000 752^***^ (0.000 062 7)	0.000 752^***^ (0.000 062 7)	0.000 752^***^ (0.000 062 8)	0.000 752^***^ (0.000 062 7)	0.000 752^***^ (0.000 062 7)
Age (centered)	-0.0765^***^ (0.006 58)	-0.0766^***^ (0.006 58)	-0.0766^***^ (0.006 58)	-0.0766^***^ (0.006 58)	-0.0766^***^ (0.006 58)	-0.0766^***^ (0.006 58)	-0.0765^***^ (0.006 57)
Gender (woman)	-0.236^***^ (0.0436)	-0.236^***^ (0.0436)	-0.236^***^ (0.0436)	-0.236^***^ (0.0436)	-0.236^***^ (0.0436)	-0.236^***^ (0.0436)	-0.236^***^ (0.0436)
Primary education	-0.122 (0.0804)	-0.122 (0.0804)	-0.122 (0.0805)	-0.122 (0.0805)	-0.122 (0.0804)	-0.122 (0.0804)	-0.122 (0.0804)
Secondary education	-0.257^***^ (0.0406)	-0.257^***^ (0.0405)	-0.257^***^ (0.0405)	-0.257^***^ (0.0405)	-0.257^***^ (0.0406)	-0.257^***^ (0.0406)	-0.257^***^ (0.0405)
Financial strain	-0.513^***^ (0.0495)	-0.513^***^ (0.0494)	-0.513^***^ (0.0495)	-0.513^***^ (0.0494)	-0.513^***^ (0.0494)	-0.513^***^ (0.0495)	-0.513^***^ (0.0495)
Survey wave dummy (2016)	0.0382 (0.0922)	0.0638 (0.0985)	0.0358 (0.0954)	0.0440 (0.0984)	0.0492 (0.0935)	0.0439 (0.0943)	0.0329 (0.0938)
Life expectancy (region)	-0.006 00 (0.0387)	-0.0126 (0.0409)	-0.007 50 (0.0401)	-0.008 78 (0.0406)	-0.0105 (0.0396)	-0.009 83 (0.0399)	0.000 046 0 (0.0383)
PHE (ln)	1.184^*^ (0.490)	0.769 (0.687)	1.229^*^ (0.578)	1.095^+^ (0.622)	1.009^+^ (0.574)	1.097^+^ (0.600)	1.259^*^ (0.519)
Decentralisation provision	0.367^*^ (0.160)						
Decentralisation implementation		0.341 (0.212)					
Decentralisation financing			0.0289 (0.143)				
Decentralisation public healthcare				0.171 (0.213)			
Decentralisation secondary/tertiary care					0.328^+^ (0.188)		
Decentralisation primary care						0.225 (0.188)	
Decentralisation index = 1							1.273^**^ (0.477)
Decentralisation index = 2							1.078^*^ (0.528)
Constant	-5.696 (2.929)	-1.676 (5.328)	-5.188 (4.021)	-4.268 (4.470)	-3.702 (3.846)	-4.264 (4.153)	-7.022^*^ (3.201)
Country-level variance	0.484	0.535	0.636	0.617	0.539	0.590	0.441
Region-level variance	0.175	0.175	0.175	0.175	0.175	0.175	0.175
N	71 454	71 454	71 454	71 454	71 454	71 454	71 454

Abbreviation: PHE, public health expenditure in US$1000 per capita. Standard errors in parentheses. ^+^ *P *< .1 ^*^ *P *< .05, ^**^ *P *< .01, ^***^ *P *< .001.

Turning to the control variables at individual level, we found that high age, female gender, low education, financial strain, and hampering health problems was negatively and significantly associated with both self-rated health and health system satisfaction. At the regional level, the associations between life expectancy and the dependent variables were without statistical significance in the final models, but were significantly associated with self-rated health in sensitivity analyses where public health expenditure was left out of the model. The expenditure measure at the country level had no significant associations with self-rated health but was positively and significantly associated with health system satisfaction in all models except for the implementation model.

We assessed model fit by studying reductions in unexplained variance from the baseline to the full models. For self-rated health, approximately 7% of variance was located at the country level in the baseline model; for the models included in Table 3, this figure was approximately 3%, with that reducing country-level variance by 4 percentage points or approximately 55%. The different decentralisation models all returned similar variance reductions. For health system satisfaction, the baseline model showed 21% country level variation; this was reduced to between 8% and 12% by the included models. Model 1 and Model 7, with decentralised provision and the decentralisation index as main explanatory variables, showed the highest reductions in country-level variance, with the latter model reducing variance with approximately 60%. The reduction in region-level variance was not substantial in the models which included the life expectancy variable.

## Discussion

Our study used the most recent data available on European countries, including 70 000 respondents from 20 countries, to investigate the association between allocation of authority in health policy and health system performance and outcomes. While many earlier studies, in particular of health outcomes, have measured decentralisation only in fiscal terms, we employed indicators that reflect several political dimensions of healthcare decentralisation. Focusing only on the fiscal aspect of decentralisation is likely to provide a misleading picture of the real level of autonomy in policy-making of sub national tiers of government. The decentralisation index used here, developed by Adolph et al,^7^ reflects the autonomy in health policy enjoyed by lower levels of government by capturing the allocation of responsibility for key healthcare policy tasks and policy areas. As outcome measures we employed 2 indicators that have not earlier been tested in the context of decentralisation: self-rated health and evaluation of health services. We present mixed evidence for the case of political decentralisation in healthcare. The results indicate that decentralisation has a positive and significant association with health system satisfaction, but not with self-rated health. Our findings thus only partially corroborate the earlier studies of decentralisation that has employed fiscal measures: although far from conclusive, they generally seem to lend support to the argument of fiscally decentralised health systems.^1,32^

Ironically, the arguments used to promote decentralisation are also the same put forward by the centralisers. The supporters of centralisation maintain that it will achieve allocative efficiency, allowing for the distribution of funds across a national population according to need, and benefit from economies of scale.^55^ This argument against political decentralisation rests on an economic perspective, focusing on the inefficiency and duplication of having multiple small service providers. Decentralisation may increase overall costs if it leads to the creation of an additional layer of administrative governance. Furthermore, spreading the decision capacity to several decentralised units may create problems in coordinating these units, and planning of investments and development of treatment facilities may consequently become sub-optimal. If steering ambitions from the central level face opposition by strong decentralised units it may also create difficulties in imposing common standards and create transparency.^56^

A central argument made against political decentralisation on democratic grounds focuses on the degree of inequity that can accompany variation in service provision. Since health services tend to cost more in remote and sparsely populated areas, equity issues may arise in relation to decentralisation, especially if decentralisation of financing is involved. Furthermore, inequity in access could increase or decrease because of local decision-makers’ choice of resource use, even if financing is distributed equitably. Inequalities between areas may also result from different capacities to use resources efficiently, or local priorities may be at odds with national policy priorities or given local responsibilities.^57^ Although the acceptance of local differences is an inherent (but not always explicit) consequence of decentralisation and a requirement for a number of listed benefits (adjusting to local needs, local level experimentation, etc), the occurrence of equity problems often gives rise to public or political pressure for standardisation and equalisation across units. Recentralising can provide better possibilities for setting standards and holding delivery organisations accountable to uniform principles.^56^ Inequity has been the most frequent concern among the studies reporting negative or ambiguous effects from healthcare decentralisation.^57-59^ A recent systematic review of the literature concluded that the implications of decentralised governance on health-related equity are varied and depend on pre-existing socio-economic and organisational context, the form of decentralisation implemented and the complementary mechanisms implemented alongside decentralisation.^60^

Considering that the most appropriate level for the decentralisation of health policy is still an important unresolved issue in the research literature, it is surprising that so little attention has been paid to the evaluation of decentralisation in European healthcare systems.^1,32^ Despite its widespread adoption, decentralisation has not been well defined neither conceptually nor organisationally. Rather than representing a single strategy, decentralisation is instead a wide umbrella under which many different, often conflicting theories and approaches can be found. Many of the promises of decentralisation have therefore proven difficult to materialise, and the idea of decentralisation has thus spread across countries without much empirical evidence. This is similar with many areas of health policy, where it often has been more the case of the transfer of an idea or label rather than the transfer of substantial knowledge or reform. This underscores the importance of distinguishing between different types of decentralisation when addressing this concept: by calling everything that reduces the power of the central state for “decentralisation,” we risk lumping together processes with distinct political origins and policy consequences.^7^ It is this lack of analytic criteria that represents the key challenge in determining the outcome of decentralisation: dimensions such as responsibility, autonomy, power and accountability are difficult to quantify, and fiscal indicators can often be misleading measures of power and authority.^61^

Our study has some possible limitations that should be noted. First, it is important to consider possible artefacts due to cultural differences in European countries. Health expectations may vary according to culture, and direct cultural comparisons of self-rated health outcomes should therefore in general be made with caution. However, the strength of this study is that all questions are collected from the same survey, asking the same questions with the same period of time.^24^

Secondly, the indicator of health might be sensitive to the cut off point on the health scale. Defining ‘fair health’ as ‘good health’ could change the between-country differences, as the category ‘fair’ may not be strictly comparable between countries. However, sensitivity analyses showed that the results changed only marginally when ‘fair’ was defined as ‘good health.’ Moreover, defining ‘fair’ as ‘poor health’ has become more or less the standard procedure within social epidemiology and we have mainly done this for comparable reasons.^24^

A third limitation is the problem of omitted variables. Obviously, both health status and health services satisfaction are related to many other factors that are not accounted for in our analyses. One evident example is the social determinants of health; ie, the conditions in which people are born, grow, live, work and age, and which are shaped by the distribution of money, power and resources at global, national and local levels.^62^ Our model controls for key socio-economic status indicators at the individual level and for life expectancy at the regional level, which may indirectly capture some of the impact of socio-economic determinants of health. We also estimated fixed effects-models with dummy variables included for regions for each country, which picks up regional variation not explained in our model that could potentially reflect social determinants, but this did not change the results. A study of decentralisation and health system performance and outcomes should include better controls for aspects such as housing, economic and social relationships, education, employment and work conditions, and the next step would be to gather such information to include in future analyses. Our model also lacks important regional health system characteristics that could influence perceived health status and health services quality, such as regional financing, private provision, waiting times and organisational solutions. Some of this variation would be picked up by the fixed effects approach, but a more detailed way of specifying these characteristics would off course be desired.

Finally, and needless to say, the complex nature of decentralisation is difficult to capture in a quantitative study. There are numerous contextual factors that affect health system reforms such as decentralisation, and the impact of these changes therefore results from the dynamic interaction of multiple subsystems.^63^ Our indicator of decentralisation does not allow us to distinguish between different types of decentralisation, such as for instance deconcentration, delegation and devolution. However, the measure of Adolph et al^7^ was specifically developed to capture the allocation of authorityin European health policy, and is therefore a natural starting point when seeking to investigate decentralisation in the context of European health system performance and outcomes. Some may argue that alternative indicators are better to classify and measure decentralisation. One possible approach would be to employ the “decision space” approach introduced by Bossert,^64^ which is gaining increasing popularity. Decision space is defined as the range of effective choice that is allowed by the central authorities (the principal) to be utilised by local authorities (the agents). The framework was introduced for developing countries, and has consequently – with some exceptions^65^– so far been restricted to analyses of such countries,^66^ but future studies could look into the possibilities of adapting it to cross-national comparisons of European countries. Ideally, a measure of decentralisation could also take into account the process of which decentralisation was implemented, since top-down approaches of decentralisation may be less successful, especially if regions were not looking forward to manage the health system, and if they lack the financial and human resources to manage them effectively.

## Conclusion

The concept of decentralisation has emerged as a cornerstone of health policy reforms in many European countries.^2-4^ To date, the relationship between decentralisation and health system performance and outcomes has not been investigated in European countries using cross-national data. The existing evidence has therefore been insufficient to draw firm conclusions about whether countries with more decentralised healthcare systems have better health outcomes and performance. Our study represents the first attempt to test the impact of decentralisation in European health systems on health status and patient satisfaction, but fails to provide clear support for decentralised health systems. While decentralisation in theory may lead to higher equity, efficiency, quality, access to healthcare services, and thereby better population health, it has proven difficult to materialise many of these promises. There is consequently a need for more empirical studies to document the likely effects of decentralisation in healthcare, in order to guide decision-makers towards policy choices that are appropriate. The main explanatory variables in our study are located at the country level, generated and conceptualised based on classifications from the decentralisation literature.^7^ Subsequently, the variance of these key variables is low, and by being limited to European healthcare systems, we are unable to include any further control variables at the country level. However, we argue that this study represents a novel point of departure for investigating the effect of healthcare decentralisation, using the best data available for this purpose. Future research would be enhanced by utilising richer country-level decentralisation data in a multilevel framework. New studies based on other data sources that are better able to account for important regional, provider and health system factors, the social determinants of health, and the timing and style of decentralisation reforms might well end up with different conclusions.

## Ethical issues

This study was exempted from the requirement for approval by the responsible ethics committee because it was a retrospective study of anonymised population surveys.

## Competing interests

Authors declare that they have no competing interests.

## Authors’ contributions

HTR had the main responsibility for the data analyses, both authors contributed in writing the manuscript.

## Endnotes


[1] The HiT-profiles can be accessed here: http://www.euro.who.int/en/about-us/partners/observatory/publications/health-system-reviews-hits.

[2] We also estimated models with household income (decentiles) instead of the financial strain-variable, and got similar results. Regardless, the decentralisation variables at country level remained unchanged. Cross tabulations show a close correspondence between household income and financial strain, but N drops quite drastically when using the former.


## Supplementary files

Supplementary file 1 contains Tables S1-S8.

## Key Messages

**Implications for policy makers**

- Despite its widespread adoption, many of the promises of decentralisation have proven difficult to materialise.
- The idea of decentralisation has thus spread across countries without much empirical evidence.
- Our study fails to provide policy-makers with clear empirical evidence on the effects of decentralisation on health system performance.

**Implications for public**

The public perceptions of their health and health service satisfaction are important. Citizens are beneficiaries and actors in health systems, and their opinions can be important in shaping health policies. They can provide feedback on the quality and responsiveness of services, and may bring legitimacy and accountability to the policy-making process.

## References

[1] Jiménez-Rubio D. Is fiscal decentralisation good for your health? Evidence from a panel of OECD countries. HEDG Working paper 10/30. https://ideas.repec.org/p/yor/hectdg/10-30.html. Published 2010.

[2] Saltman RB, Bankauskaite V (2006). Conceptualizing decentralization in European health systems: a functional perspective. Health Econ Policy Law.

[3] Saltman RB, Figueras J. European Health Care Reform: Analysis of Current Strategies. Copenhagen: World Health Organization, Regional Office for Europe; 1997.

[4] Saltman RB, Figueras J (1998). Analyzing the evidence on European health care reforms: experience in western European health care systems suggests lessons for reform in the United States, according to a major international comparison by the World Health Organization. Health Aff (Millwood).

[5] Dwicaksono A, Fox AM (2018). Does decentralization improve health system performance and outcomes in low-and middle-income countries? a systematic review of evidence from quantitative studies. Milbank Q.

[6] Cobos Muñoz D, Merino Amador P, Monzon Llamas L, Martinez Hernandez D, Santos Sancho JM (2017). Decentralization of health systems in low and middle income countries: a systematic review. Int J Public Health.

[7] Adolph C, Greer SL, Massard da Fonseca E (2012). Allocation of authority in European health policy. Soc Sci Med.

[8] Bhatia M, Rannan-Eliya R, Somanathan A, Huq MN, Pande BR, Chuluunzagd B (2009). Public views of health system issues in four Asian countries. Health Aff (Millwood).

[9] Blendon RJ, Kim M, Benson JM (2001). The public versus the World Health Organization on health system performance. Health Aff (Millwood).

[10] Munro N, Duckett J (2016). Explaining public satisfaction with health-care systems: findings from a nationwide survey in China. Health Expect.

[11] Busse R, Valentine N, Lessof S, Prasad A, van Ginneken E. Being responsive to citizens’ expectations: the role of health services in responsiveness and satisfaction. In: McKee M, Figueras J, eds. Health Systems, Health, Wealth and Societal Well-being: Assessing the Case for Investing in Health Systems. Maidenhead: Open University Press; 2011:175-208.

[12] Bleich SN, Özaltin E, Murray CK (2009). How does satisfaction with the health-care system relate to patient experience?. Bull World Health Organ.

[13] Papanicolas I, Cylus J, Smith PC (2013). An analysis of survey data from eleven countries finds that ‘satisfaction’ with health system performance means many things. Health Aff (Millwood).

[14] Borisova LV, Martinussen PE, Rydland HT, Stornes P, Eikemo TA (2017). Public evaluation of health services across 21 European countries: the role of culture. Scand J Public Health.

[15] Footman K, Roberts B, Mills A, Richardson E, McKee M (2013). Public satisfaction as a measure of health system performance: a study of nine countries in the former Soviet Union. Health Policy.

[16] Munro N, Duckett J (2016). Explaining public satisfaction with health-care systems: findings from a nationwide survey in China. Health Expect.

[17] Papanicolas I, Cylus J, Smith PC (2013). An analysis of survey data from eleven countries finds that ‘satisfaction’ with health system performance means many things. Health Aff (Millwood).

[18] Wendt C, Kohl J, Mischke M, Pfeifer M (2009). How do Europeans perceive their healthcare system? patterns of satisfaction and preference for state involvement in the field of healthcare. Eur Sociol Rev.

[19] Falconer J, Quesnel-Vallée A (2017). Pathway from poor self-rated health to mortality: explanatory power of disease diagnosis. Soc Sci Med.

[20] Au N, Johnston DW (2014). Self-assessed health: what does it mean and what does it hide?. Soc Sci Med.

[21] Dalstra JA, Kunst AE, Borrell C (2005). Socioeconomic differences in the prevalence of common chronic diseases: an overview of eight European countries. Int J Epidemiol.

[22] Idler EL, Benyamini Y (1997). Self-rated health and mortality: a review of twenty-seven community studies. J Health Soc Behav.

[23] Bombak AE (2013). Self-rated health and public health: a critical perspective. Front Public Health.

[24] Eikemo TA, Bambra C, Judge K, Ringdal K (2008). Welfare state regimes and differences in self-perceived health in Europe: a multilevel analysis. Soc Sci Med.

[25] Bennett IM, Chen J, Soroui JS, White S (2009). The contribution of health literacy to disparities in self-rated health status and preventive health behaviors in older adults. Ann Fam Med.

[26] van der Wel KA, Saltkjel T, Chen WH, Dahl E, Halvorsen K (2018). European health inequality through the ‘Great Recession’: social policy matters. Sociol Health Illn.

[27] Delaruelle K, Buffel V, Bracke P (2018). The reversal of the gender gap in education: what does it mean for gender differences in the relationship between education and health. Eur Sociol Rev.

[28] Delaruelle K, van de Werfhorst H, Bracke P (2019). Do comprehensive school reforms impact the health of early school leavers? results of a comparative difference-in-difference design. Soc Sci Med.

[29] Oates WE. Fiscal Federalism. New York: Harcourt Brace Jovanovich; 1972.

[30] Gravelle H (2003). A Comment on Weale’s Paper from an Economic Perspective.

[31] Khaleghian P. Decentralization and Public Services: The Case of Immunization. Washington, DC: The World Bank; 2003. 10.1016/j.socscimed.2003.10.013 15087152

[32] Rubio DJ (2011). The impact of decentralization of health services on health outcomes: evidence from Canada. Appl Econ.

[33] Saltman RB, Bankauskaite V, Vrangbæk K. Decentralization in Health Care: Strategies and Outcomes. Maidenhead: McGraw-Hill, Open University Press; 2007.

[34] Habibi N, Huang C, Miranda D, et al. Decentralization in Argentina. New Haven, CT: Yale University, Economic Growth Center; 2001.

[35] Yee E (2001). The effects of fiscal decentralization on health care in China. University Avenue Undergraduate Journal of Economics.

[36] Jin Y, Sun R (2011). Does fiscal decentralization improve healthcare outcomes? empirical evidence from China. Public Finance and Management.

[37] Asfaw A, Frohberg K, James KS, Jütting J (2007). Fiscal decentralization and infant mortality: empirical evidence from rural India. J Dev Areas.

[38] Samadi AH, Keshtkaran A, Kavosi Z, Vahedi S (2013). The effect of fiscal decentralization on under-five mortality in Iran: a panel data analysis. Int J Health Policy Manag.

[39] Cantarero D, Pascual M (2008). Analysing the impact of fiscal decentralization on health outcomes: empirical evidence from Spain. Appl Econ Lett.

[40] Robalino DA, Picazo OF, Voetberg A. Does Fiscal Decentralization Improve Health Outcomes? Evidence from a Cross-Country Analysis. Washington: The World Bank; 2001.

[41] Hodge A, Firth S, Jimenez-Soto E, Trisnantoro L (2015). Linkages between decentralisation and inequalities in neonatal health: evidence from Indonesia. J Dev Stud.

[42] Kengia JT, Igarashi I, Kawabuchi K (2013). Effectiveness of health sector reforms in reducing disparities in utilization of skilled birth attendants in Tanzania. Tohoku J Exp Med.

[43] Vargas Bustamante A (2010). The tradeoff between centralized and decentralized health services: evidence from rural areas in Mexico. Soc Sci Med.

[44] Khaleghian P (2004). Decentralization and public services: the case of immunization. Soc Sci Med.

[45] Kruse I, Pradhan M, Sparrow R (2012). Marginal benefit incidence of public health spending: evidence from Indonesian sub-national data. J Health Econ.

[46] Maharani A, Tampubolon G (2014). Has decentralisation affected child immunisation status in Indonesia?. Glob Health Action.

[47] Faguet JP, Sánchez F (2014). Decentralization and access to social services in Colombia. Public Choice.

[48] European Social Survey. European Social Survey Cumulative File, ESS 7-82016. https://www.europeansocialsurvey.org/.

[49] Eikemo TA, Bambra C, Huijts T, Fitzgerald R (2016). The first pan-European sociological health inequalities survey of the general population: the European Social Survey rotating module on the social determinants of health. Eur Sociol Rev.

[50] OECD. OECD Health Statistics 2018: Health Care Resources. http://www.oecd.org/els/health-systems/health-data.htm. Published 2018.

[51] OECD. OECD Health Statistics 2018: Health Expenditure and Financing. http://www.oecd.org/els/health-systems/health-data.htm. Published 2018.

[52] OECD. OECD Regions and Cities Statistics 2018: Subnational Government Expenditure by category. https://www.oecd-ilibrary.org/governance/oecd-regions-and-cities-at-a-glance-2018/subnational-government-expenditure-by-category_reg_cit_glance-2018-42-en. Published 2018.

[53] European Observatory on Health Systems and Policies. Health in Transition series. http://www.euro.who.int/en/about-us/partners/observatory/publications/health-system-reviews-hits. Published 2017.

[54] Wendt C (2009). Mapping European healthcare systems: a comparative analysis of financing, service provision and access to healthcare. J Eur Soc Policy.

[55] Bremner J (2011). The complexities of decentralization. Euro Observer.

[56] Vrangbæk K. Key factors in assessing decentralization and recentralization in health systems. In: Saltman RB, Bankauskaite V, Vrangbæk K, eds. Decentralization in Health Care: Strategies and Outcomes. Maidenhead: McGraw-Hill, Open University Press; 2007:63.

[57] Koivusalo M, Wyss K, Santana P. Effects of decentralization and recentralization on equity dimensions of health systems. In: Saltman RB, Bankauskaite V, Vrangbæk K, eds. Decentralization in Health Care: Strategies and Outcomes. Maidenhead: McGraw-Hill, Open University Press; 2007:189.

[58] Collins C, Green A (1994). Decentralization and primary health care: some negative implications in developing countries. Int J Health Serv.

[59] Jommi C, Fattore G (2003). Regionalization and drugs cost-sharing in the Italian NHS. Euro Observer.

[60] Sumah AM, Baatiema L, Abimbola S (2016). The impacts of decentralisation on health-related equity: a systematic review of the evidence. Health Policy.

[61] Bankauskaite V, Saltman RB. Central issues in the decentralization debate. In: Saltman RB, Bankauskaite V, Vrangbæk K, eds. Decentralization in Health Care: Strategies and Outcomes. Maidenhead: McGraw-Hill, Open University Press; 2007:9.

[62] World Health Organization (WHO). Progressing the Sustainable Development Goals through Health in All Policies: Case Studies from Around the World. Adelaide: Government of South Australia; 2017.

[63] Atun R (2012). Health systems, systems thinking and innovation. Health Policy Plan.

[64] Bossert T (1998). Analyzing the decentralization of health systems in developing countries: decision space, innovation and performance. Soc Sci Med.

[65] Marchildon G, Bossert TJ. Federalism and Decentralization in Health Care: A Decision Space Approach. Toronto: University of Toronto Press; 2018.

[66] Roman TE, Cleary S, McIntyre D (2017). Exploring the functioning of decision space: a review of the available health systems literature. Int J Health Policy Manag.

