# Supplementary material for: Is a Decentralised Health Policy Associated With Better Self-rated Health and Health Services Evaluation? A Comparative Study of European Countries
Source: Int J Health Policy Manag. 2020 Feb 22;10(2):55–66. doi: 10.34172/ijhpm.2020.13 (PMC7947669; doi:10.34172/ijhpm.2020.13)
Supplement: Supplementary file 1 — contains Tables S1-S8. [file ijhpm-10-55-Supp1.pdf]

Supplementary file 1. Results from Sensitivity Analyses

Table S1. Sensitivity analyses

**Provision**	**Self-rated Health**				**Health System Satisfaction**			
	**Model 1**	**Model 2**	**Model 3**	**Model 4**	**Model 5**	**Model 6**	**Model 7**	**Model 8**
Age (squared)	0.000194*** (0.0000558)	0.000194*** (0.0000558)	0.000194*** (0.0000559)	0.000194*** (0.0000558)	0.000752*** (0.0000628)	0.000752*** (0.0000627)	0.000752*** (0.0000628)	0.000751*** (0.0000627)
Age (centered)	-0.0673*** (0.00527)	-0.0673*** (0.00527)	-0.0673*** (0.00527)	-0.0673*** (0.00525)	-0.0765*** (0.00658)	-0.0765*** (0.00658)	-0.0765*** (0.00658)	-0.0765*** (0.00657)
Gender (woman)	-0.167** (0.0540)	-0.167** (0.0538)	-0.167** (0.0540)	-0.167** (0.0540)	-0.236*** (0.0435)	-0.236*** (0.0435)	-0.236*** (0.0435)	-0.236*** (0.0434)
Primary education	-0.829*** (0.0655)	-0.829*** (0.0654)	-0.829*** (0.0653)	-0.829*** (0.0652)	-0.122 (0.0804)	-0.122 (0.0805)	-0.122 (0.0804)	-0.122 (0.0805)
Secondary education	-0.449*** (0.0637)	-0.449*** (0.0636)	-0.450*** (0.0639)	-0.450*** (0.0638)	-0.257*** (0.0406)	-0.257*** (0.0405)	-0.257*** (0.0405)	-0.256*** (0.0406)
Financial strain	-1.146*** (0.0692)	-1.146*** (0.0695)	-1.146*** (0.0692)	-1.146*** (0.0692)	-0.513*** (0.0495)	-0.513*** (0.0495)	-0.513*** (0.0495)	-0.513*** (0.0495)
Survey wave dummy (2016)	-0.0723 (0.0512)	-0.0736 (0.0508)	-0.0751 (0.0510)	-0.0616 (0.0573)	0.108 (0.0937)	0.116 (0.0956)	0.111 (0.0950)	0.0912 (0.107)
Life exp. (region)	0.0628* (0.0248)	0.0661* (0.0314)	0.0565* (0.0257)	0.0620* (0.0253)	0.00127 (0.0365)	-0.00125 (0.0384)	0.000736 (0.0359)	0.0101 (0.0373)
Decentralized health provision	-0.0218 (0.0927)	-0.0246 (0.0917)	-0.0648 (0.0993)	-0.0191 (0.0918)	0.456*** (0.132)	0.477*** (0.130)	0.497** (0.193)	0.461*** (0.121)
OOP (% of THE)		0.00529 (0.0158)				-0.0444 (0.0486)		
GPs (per 1000 inh.)			0.425 (0.398)				-0.413 (1.083)	
Access regulation = 1				-0.0573 (0.0603)				0.152 (0.109)
Access regulation = 2				-0.145 (0.161)				-0.285 (0.323)
Access regulation = 3				-0.0931 (0.150)				-0.371 (0.229)
Constant	-1.596 (2.001)	-1.953 (2.792)	-1.336 (1.963)	-1.438 (2.027)	2.806 (2.872)	3.806 (3.419)	3.093 (3.099)	2.356 (2.990)
Country-level variance	0.135*** (0.0408)	0.132** (0.0421)	0.129*** (0.0354)	0.140*** (0.0424)	0.897 (0.262)	0.775 (0.259)	1.018 (0.575)	0.797 (0.255)
Region-level variance	0.0381** (0.0127)	0.0381** (0.0127)	0.0378** (0.0126)	0.0377** (0.0123)	0.172*** (0.0417)	0.174*** (0.0419)	0.172*** (0.0418)	0.169*** (0.0417)
N	71868	71868	71868	71868	71454	71454	71454	71454

Abbreviations: OOP, out-of-pocket payment; GPs, general practitioners.
Standard errors in parentheses. $^{+}$ $P < .1$, * $P < .05$, ** $P < .01$, *** $P < .001$.

Table S2. Sensitivity analyses

Implementation	Self-Rated Health				Health System Satisfaction			
	**Model 1**	**Model 2**	**Model 3**	**Model 4**	**Model 5**	**Model 6**	**Model 7**	**Model 8**
Age (squared)	0.000194*** (0.0000558)	0.000193*** (0.0000558)	0.000193*** (0.0000559)	0.000193*** (0.0000557)	0.000752*** (0.0000628)	0.000752*** (0.0000627)	0.000752*** (0.0000628)	0.000752*** (0.0000627)
Age (centered)	-0.0673*** (0.00527)	-0.0673*** (0.00527)	-0.0672*** (0.00527)	-0.0673*** (0.00525)	-0.0765*** (0.00658)	-0.0765*** (0.00658)	-0.0766*** (0.00658)	-0.0765*** (0.00657)
Gender (woman)	-0.167** (0.0540)	-0.167** (0.0539)	-0.167** (0.0541)	-0.167** (0.0541)	-0.236*** (0.0435)	-0.236*** (0.0435)	-0.236*** (0.0435)	-0.236*** (0.0434)
Primary education	-0.829*** (0.0655)	-0.829*** (0.0655)	-0.828*** (0.0652)	-0.829*** (0.0653)	-0.122 (0.0803)	-0.122 (0.0804)	-0.122 (0.0804)	-0.122 (0.0804)
Secondary education	-0.449*** (0.0637)	-0.449*** (0.0636)	-0.450*** (0.0638)	-0.450*** (0.0638)	-0.257*** (0.0405)	-0.257*** (0.0405)	-0.257*** (0.0406)	-0.256*** (0.0406)
Financial strain	-1.146*** (0.0692)	-1.146*** (0.0694)	-1.146*** (0.0691)	-1.146*** (0.0692)	-0.513*** (0.0494)	-0.513*** (0.0495)	-0.513*** (0.0495)	-0.513*** (0.0494)
Survey wave dummy (2016)	-0.0719 (0.0513)	-0.0731 (0.0508)	-0.0742 (0.0508)	-0.0616 (0.0571)	0.109 (0.0933)	0.115 (0.0949)	0.104 (0.0958)	0.0911 (0.107)
Life exp. (region)	0.0604* (0.0276)	0.0633 (0.0329)	0.0563* (0.0278)	0.0604* (0.0280)	-0.00890 (0.0401)	-0.0119 (0.0413)	-0.00994 (0.0372)	-0.000958 (0.0404)
Decentralized health implementation	0.0163 (0.0708)	0.0183 (0.0705)	0.00971 (0.0734)	0.0109 (0.0702)	0.501** (0.154)	0.476** (0.154)	0.475*** (0.139)	0.486*** (0.147)
OOP (% of THE)		0.00519 (0.0165)				-0.0317 (0.0453)		
GPs (per 1,000 inh.)			0.320 (0.343)				0.827 (0.635)	
Access regulation = 1				-0.0543 (0.0605)				0.155 (0.107)
Access regulation = 2				-0.142 (0.156)				-0.195 (0.305)
Access regulation = 3				-0.0883 (0.142)				-0.302 (0.200)
Constant	-1.473 (2.196)	-1.805 (2.876)	-1.385 (2.131)	-1.369 (2.209)	3.804 (3.041)	4.682 (3.466)	3.264 (3.051)	3.394 (3.119)
Country-level variance	0.136** (0.0450)	0.133** (0.0479)	0.132** (0.0438)	0.140** (0.0463)	0.679 (0.147)	0.636 (0.150)	0.471* (0.171)	0.619* (0.142)
Region-level variance	0.0381** (0.0127)	0.0381** (0.0127)	0.0379** (0.0126)	0.0377** (0.0123)	0.173*** (0.0419)	0.174*** (0.0421)	0.173*** (0.0419)	0.170*** (0.0419)
N	71868	71868	71868	71868	71454	71454	71454	71454

Standard errors in parentheses. + $P < .1$, * $P < .05$, ** $P < .01$, *** $P < .001$.

Table S3. Sensitivity analyses

Financing	Self-Rated Health				Health System Satisfaction			
	**Model 1**	**Model 2**	**Model 3**	**Model 4**	**Model 5**	**Model 6**	**Model 7**	**Model 8**
Age (squared)	0.000194*** (0.0000558)	0.000194*** (0.0000557)	0.000193*** (0.0000558)	0.000194*** (0.0000557)	0.000752*** (0.0000628)	0.000752*** (0.0000627)	0.000752*** (0.0000628)	0.000752*** (0.0000627)
Age (centered)	-0.0673*** (0.00526)	-0.0673*** (0.00526)	-0.0672*** (0.00526)	-0.0673*** (0.00525)	-0.0765*** (0.00658)	-0.0765*** (0.00658)	-0.0765*** (0.00658)	-0.0765*** (0.00657)
Gender (woman)	-0.167** (0.0540)	-0.167** (0.0539)	-0.167** (0.0540)	-0.167** (0.0540)	-0.236*** (0.0435)	-0.236*** (0.0435)	-0.236*** (0.0435)	-0.236*** (0.0434)
Primary education	-0.828*** (0.0656)	-0.829*** (0.0656)	-0.828*** (0.0652)	-0.829*** (0.0653)	-0.122 (0.0803)	-0.122 (0.0805)	-0.122 (0.0804)	-0.122 (0.0804)
Secondary education	-0.449*** (0.0638)	-0.449*** (0.0636)	-0.450*** (0.0638)	-0.450*** (0.0638)	-0.257*** (0.0405)	-0.257*** (0.0405)	-0.257*** (0.0405)	-0.256*** (0.0406)
Financial strain	-1.146*** (0.0691)	-1.146*** (0.0694)	-1.146*** (0.0691)	-1.146*** (0.0691)	-0.513*** (0.0495)	-0.513*** (0.0495)	-0.513*** (0.0494)	-0.513*** (0.0494)
Survey wave dummy (2016)	-0.0725 (0.0510)	-0.0737 (0.0505)	-0.0746 (0.0506)	-0.0619 (0.0568)	0.109 (0.0936)	0.116 (0.0957)	0.109 (0.0955)	0.0924 (0.108)
Life exp. (region)	0.0637* (0.0258)	0.0667* (0.0315)	0.0585* (0.0266)	0.0625* (0.0260)	-0.00350 (0.0386)	-0.00547 (0.0399)	-0.00355 (0.0378)	0.00397 (0.0395)
Decentralized health financing	-0.0106 (0.0609)	-0.0100 (0.0598)	-0.00937 (0.0595)	-0.00680 (0.0611)	0.150 (0.155)	0.130 (0.154)	0.150 (0.156)	0.191 (0.158)
OOP (% of THE)		0.00484 (0.0164)				-0.0385 (0.0554)		
GPs (per 1,000 inh.)			0.323 (0.344)				-0.0686 (1.045)	
Access regulation = 1				-0.0549 (0.0593)				0.148 (0.111)
Access regulation = 2				-0.143 (0.156)				-0.293 (0.356)
Access regulation = 3				-0.0890 (0.146)				-0.387 (0.267)
Constant	-1.706 (2.055)	-2.033 (2.772)	-1.547 (2.013)	-1.518 (2.063)	4.004 (3.024)	4.911 (3.536)	4.062 (3.297)	3.645 (3.155)
Country-level variance	0.135** (0.0428)	0.132** (0.0450)	0.132** (0.0423)	0.140** (0.0440)	1.113 (0.286)	1.026 (0.310)	1.139 (0.638)	0.993 (0.286)
Region-level variance	0.0381** (0.0127)	0.0381** (0.0127)	0.0379** (0.0126)	0.0377** (0.0123)	0.173*** (0.0418)	0.174*** (0.0420)	0.173*** (0.0418)	0.170*** (0.0419)
N	71868	71868	71868	71868	71454	71454	71454	71454

Standard errors in parentheses. $^{+}$ $P < .1$, * $P < .05$, ** $P < .01$, *** $P < .001$.

Table S4. Sensitivity analyses

Public Health	Self-Rated Health				Health System Satisfaction			
	Model 1	Model 2	Model 3	Model 4	Model 5	Model 6	Model 7	Model 8
Age (squared)	0.000194*** (0.0000558)	0.000194*** (0.0000558)	0.000193*** (0.0000559)	0.000194*** (0.0000557)	0.000752*** (0.0000628)	0.000752*** (0.0000627)	0.000752*** (0.0000628)	0.000752*** (0.0000627)
Age (centered)	-0.0673*** (0.00526)	-0.0673*** (0.00526)	-0.0672*** (0.00526)	-0.0673*** (0.00525)	-0.0765*** (0.00658)	-0.0765*** (0.00658)	-0.0765*** (0.00658)	-0.0765*** (0.00657)
Gender (woman)	-0.167** (0.0540)	-0.167** (0.0539)	-0.167** (0.0540)	-0.167** (0.0540)	-0.236*** (0.0435)	-0.236*** (0.0435)	-0.236*** (0.0435)	-0.236*** (0.0434)
Primary education	-0.828*** (0.0657)	-0.829*** (0.0657)	-0.828*** (0.0653)	-0.828*** (0.0654)	-0.122 (0.0804)	-0.122 (0.0805)	-0.122 (0.0804)	-0.122 (0.0805)
Secondary education	-0.450*** (0.0638)	-0.449*** (0.0636)	-0.450*** (0.0639)	-0.450*** (0.0638)	-0.257*** (0.0405)	-0.257*** (0.0405)	-0.257*** (0.0406)	-0.256*** (0.0406)
Financial strain	-1.146*** (0.0693)	-1.146*** (0.0695)	-1.146*** (0.0692)	-1.146*** (0.0693)	-0.513*** (0.0495)	-0.513*** (0.0495)	-0.513*** (0.0494)	-0.513*** (0.0495)
Survey wave dummy (2016)	-0.0729 (0.0513)	-0.0737 (0.0509)	-0.0751 (0.0510)	-0.0621 (0.0574)	0.109 (0.0936)	0.115 (0.0955)	0.107 (0.0958)	0.0919 (0.108)
Life exp. (region)	0.0659* (0.0264)	0.0679* (0.0313)	0.0611* (0.0266)	0.0651* (0.0269)	-0.00442 (0.0396)	-0.00629 (0.0407)	-0.00430 (0.0383)	0.00359 (0.0403)
Decentralized public health	-0.0348 (0.0746)	-0.0310 (0.0783)	-0.0391 (0.0712)	-0.0368 (0.0756)	0.351^{+} (0.197)	0.307 (0.232)	0.346^{+} (0.193)	0.368^{+} (0.188)
OOP (% of THE)		0.00385 (0.0168)				-0.0328 (0.0559)		
GPs (per 1,000 inh.)			0.332 (0.355)				0.211 (0.874)	
Access regulation = 1				-0.0572 (0.0611)				0.151 (0.110)
Access regulation = 2				-0.144 (0.162)				-0.280 (0.336)
Access regulation = 3				-0.0892 (0.152)				-0.379 (0.243)
Constant	-1.834 (2.090)	-2.077 (2.747)	-1.706 (2.010)	-1.677 (2.112)	3.676 (3.010)	4.516 (3.548)	3.504 (3.207)	3.277 (3.134)
Country-level variance	0.134*** (0.0382)	0.131** (0.0412)	0.129*** (0.0376)	0.138*** (0.0391)	0.965 (0.287)	0.918 (0.322)	0.894 (0.561)	0.850 (0.291)
Region-level variance	0.0381** (0.0127)	0.0381** (0.0127)	0.0379** (0.0126)	0.0377** (0.0123)	0.173*** (0.0418)	0.174*** (0.0420)	0.173*** (0.0418)	0.170*** (0.0419)
N	71868	71868	71868	71868	71454	71454	71454	71454

Standard errors in parentheses. $^{+}$ $P < .1$, * $P < .05$, ** $P < .01$, *** $P < .001$.

Table S5. Sensitivity analyses

Secondary/ Tertiary	Self-Rated Health				Health System Satisfaction			
	Model 1	Model 2	Model 3	Model 4	Model 5	Model 6	Model 7	Model 8
Age (squared)	0.000194*** (0.0000559)	0.000194*** (0.0000559)	0.000193*** (0.0000559)	0.000194*** (0.0000558)	0.000752*** (0.0000628)	0.000752*** (0.0000627)	0.000752*** (0.0000628)	0.000751*** (0.0000627)
Age (centered)	-0.0673*** (0.00527)	-0.0673*** (0.00527)	-0.0673*** (0.00527)	-0.0673*** (0.00525)	-0.0765*** (0.00658)	-0.0765*** (0.00658)	-0.0765*** (0.00658)	-0.0765*** (0.00657)
Gender (woman)	-0.167** (0.0540)	-0.167** (0.0538)	-0.167** (0.0540)	-0.167** (0.0540)	-0.236*** (0.0435)	-0.236*** (0.0435)	-0.236*** (0.0435)	-0.236*** (0.0434)
Primary education	-0.829*** (0.0655)	-0.829*** (0.0655)	-0.828*** (0.0652)	-0.829*** (0.0653)	-0.122 (0.0803)	-0.122 (0.0805)	-0.122 (0.0803)	-0.122 (0.0804)
Secondary education	-0.449*** (0.0638)	-0.449*** (0.0636)	-0.450*** (0.0638)	-0.450*** (0.0638)	-0.257*** (0.0405)	-0.257*** (0.0405)	-0.257*** (0.0405)	-0.256*** (0.0406)
Financial strain	-1.146*** (0.0692)	-1.146*** (0.0695)	-1.146*** (0.0692)	-1.146*** (0.0692)	-0.513*** (0.0495)	-0.513*** (0.0495)	-0.513*** (0.0494)	-0.513*** (0.0495)
Survey wave dummy (2016)	-0.0725 (0.0513)	-0.0737 (0.0508)	-0.0750 (0.0508)	-0.0617 (0.0572)	0.109 (0.0934)	0.117 (0.0953)	0.108 (0.0954)	0.0918 (0.107)
Life exp. (region)	0.0634* (0.0255)	0.0666* (0.0320)	0.0596* (0.0256)	0.0626* (0.0259)	-0.00453 (0.0373)	-0.00794 (0.0393)	-0.00450 (0.0362)	0.00361 (0.0377)
Decentralized sec./ter. care	-0.00906 (0.0749)	-0.0109 (0.0734)	-0.0298 (0.0799)	-0.00959 (0.0739)	0.497** (0.175)	0.494** (0.166)	0.494* (0.204)	0.492** (0.165)
OOP (% of THE)		0.00500 (0.0162)				-0.0407 (0.0465)		
GPs (per 1,000 inh.)			0.352 (0.383)				0.0432 (0.932)	
Access regulation = 1				-0.0561 (0.0604)				0.151 (0.110)
Access regulation = 2				-0.147 (0.158)				-0.265 (0.333)
Access regulation = 3				-0.0934 (0.147)				-0.361 (0.234)
Constant	-1.672 (2.037)	-2.024 (2.811)	-1.616 (1.954)	-1.513 (2.052)	3.459 (2.856)	4.509 (3.412)	3.427 (3.067)	3.071 (2.958)
Country-level variance	0.136** (0.0417)	0.132** (0.0435)	0.131*** (0.0396)	0.140** (0.0433)	0.825 (0.191)	0.730 (0.189)	0.813 (0.367)	0.738 (0.193)
Region-level variance	0.0381** (0.0127)	0.0381** (0.0127)	0.0379** (0.0126)	0.0377** (0.0123)	0.173*** (0.0419)	0.174*** (0.0420)	0.173*** (0.0419)	0.170*** (0.0419)
N	71868	71868	71868	71868	71454	71454	71454	71454

Standard errors in parentheses. + $P < .1$, * $P < .05$, ** $P < .01$, *** $P < .001$.

Table S6. Sensitivity analyses

Primary healthcare								
	Self-rated health				Health system satisfaction			
	Model 1	Model 2	Model 3	Model 4	Model 5	Model 6	Model 7	Model 8
Age (squared)	0.000193*** (0.0000558)	0.000193*** (0.0000558)	0.000193*** (0.0000559)	0.000193*** (0.0000557)	0.000752*** (0.0000628)	0.000752*** (0.0000627)	0.000752*** (0.0000628)	0.000752*** (0.0000627)
Age (centered)	-0.0673*** (0.00526)	-0.0673*** (0.00526)	-0.0672*** (0.00526)	-0.0673*** (0.00525)	-0.0765*** (0.00658)	-0.0765*** (0.00658)	-0.0765*** (0.00658)	-0.0765*** (0.00657)
Gender (woman)	-0.167** (0.0540)	-0.167** (0.0539)	-0.167** (0.0541)	-0.167** (0.0540)	-0.236*** (0.0435)	-0.236*** (0.0435)	-0.236*** (0.0435)	-0.236*** (0.0434)
Primary education	-0.829*** (0.0655)	-0.829*** (0.0655)	-0.828*** (0.0652)	-0.829*** (0.0653)	-0.122 (0.0803)	-0.122 (0.0805)	-0.122 (0.0803)	-0.122 (0.0804)
Secondary education	-0.449*** (0.0637)	-0.449*** (0.0636)	-0.450*** (0.0638)	-0.450*** (0.0638)	-0.257*** (0.0405)	-0.257*** (0.0405)	-0.257*** (0.0405)	-0.256*** (0.0406)
Financial strain	-1.146*** (0.0691)	-1.146*** (0.0694)	-1.146*** (0.0691)	-1.146*** (0.0691)	-0.513*** (0.0495)	-0.513*** (0.0495)	-0.513*** (0.0494)	-0.513*** (0.0495)
Survey wave dummy (2016)	-0.0719 (0.0512)	-0.0730 (0.0506)	-0.0741 (0.0507)	-0.0608 (0.0568)	0.109 (0.0934)	0.117 (0.0954)	0.109 (0.0953)	0.0925 (0.107)
Life exp. (region)	0.0603* (0.0255)	0.0632 (0.0323)	0.0565* (0.0258)	0.0592* (0.0257)	-0.00453 (0.0380)	-0.00784 (0.0400)	-0.00458 (0.0371)	0.00362 (0.0384)
Decentralized primary care	0.0278 (0.0740)	0.0244 (0.0709)	0.0138 (0.0739)	0.0329 (0.0748)	0.380* (0.161)	0.396* (0.154)	0.384* (0.190)	0.414** (0.151)
OOP (% of THE)		0.00426 (0.0162)				-0.0432 (0.0503)		
GPs (per 1,000 inh.)			0.313 (0.343)				-0.0622 (1.050)	
Access regulation = 1				-0.0551 (0.0595)				0.149 (0.109)
Access regulation = 2				-0.154 (0.154)				-0.330 (0.339)
Access regulation = 3				-0.0994 (0.144)				-0.421^{+} (0.250)
Constant	-1.479 (2.034)	-1.786 (2.843)	-1.407 (1.960)	-1.298 (2.048)	3.678 (2.956)	4.742 (3.519)	3.726 (3.232)	3.283 (3.043)
Country-level variance	0.135** (0.0455)	0.133** (0.0474)	0.132** (0.0441)	0.140** (0.0470)	0.949 (0.219)	0.832 (0.212)	0.969 (0.451)	0.813 (0.213)
Region-level variance	0.0381** (0.0127)	0.0381** (0.0127)	0.0379** (0.0126)	0.0376** (0.0123)	0.173*** (0.0419)	0.174*** (0.0420)	0.173*** (0.0419)	0.170*** (0.0419)
N	71868	71868	71868	71868	71454	71454	71454	71454

Standard errors in parentheses. $^{+}$ $P < .1$, * $P < .05$, ** $P < .01$, *** $P < .001$.

Table S7. Sensitivity analyses

Decentralisation Index	Self-Rated Health				Health System Satisfaction			
	**Model 1**	**Model 2**	**Model 3**	**Model 4**	**Model 5**	**Model 6**	**Model 7**	**Model 8**
Age (squared)	0.000194*** (0.0000558)	0.000194*** (0.0000558)	0.000194*** (0.0000559)	0.000194*** (0.0000557)	0.000752*** (0.0000628)	0.000752*** (0.0000627)	0.000752*** (0.0000628)	0.000751*** (0.0000627)
Age (centered)	-0.0673*** (0.00526)	-0.0673*** (0.00526)	-0.0673*** (0.00526)	-0.0673*** (0.00524)	-0.0765*** (0.00658)	-0.0765*** (0.00658)	-0.0765*** (0.00658)	-0.0765*** (0.00657)
Gender (woman)	-0.167** (0.0540)	-0.167** (0.0539)	-0.167** (0.0541)	-0.167** (0.0540)	-0.236*** (0.0435)	-0.236*** (0.0435)	-0.236*** (0.0435)	-0.236*** (0.0434)
Primary education	-0.829*** (0.0653)	-0.829*** (0.0653)	-0.829*** (0.0650)	-0.829*** (0.0651)	-0.122 (0.0803)	-0.121 (0.0805)	-0.122 (0.0804)	-0.122 (0.0804)
Secondary education	-0.449*** (0.0638)	-0.449*** (0.0636)	-0.450*** (0.0639)	-0.450*** (0.0638)	-0.257*** (0.0406)	-0.257*** (0.0405)	-0.257*** (0.0405)	-0.256*** (0.0406)
Financial strain	-1.146*** (0.0692)	-1.146*** (0.0695)	-1.146*** (0.0692)	-1.146*** (0.0692)	-0.513*** (0.0495)	-0.513*** (0.0495)	-0.513*** (0.0495)	-0.513*** (0.0495)
Survey wave dummy (2016)	-0.0722 (0.0506)	-0.0734 (0.0501)	-0.0745 (0.0503)	-0.0611 (0.0561)	0.108 (0.0939)	0.116 (0.0958)	0.108 (0.0952)	0.0909 (0.108)
Life exp. (region)	0.0623* (0.0263)	0.0654* (0.0327)	0.0539 (0.0280)	0.0610* (0.0264)	0.00374 (0.0368)	0.00199 (0.0383)	0.00360 (0.0359)	0.0106 (0.0382)
Decentralisation index = 1	-0.100 (0.346)	-0.102 (0.348)	-0.206 (0.347)	-0.110 (0.349)	1.365*** (0.409)	1.422** (0.453)	1.382* (0.570)	1.219** (0.438)
Decentralisation index = 2	-0.116 (0.342)	-0.118 (0.343)	-0.192 (0.338)	-0.111 (0.339)	1.463*** (0.325)	1.469*** (0.384)	1.478*** (0.419)	1.450*** (0.323)
OOP (% of THE)		0.00508 (0.0162)				-0.0427 (0.0475)		
GPs (per 1000 inh.)			0.404 (0.385)				-0.0761 (1.059)	
Access regulation = 1				-0.0577 (0.0575)				0.151 (0.111)
Access regulation = 2				-0.152 (0.153)				-0.241 (0.355)
Access regulation = 3				-0.101 (0.140)				-0.330 (0.268)
Constant	-1.511 (2.195)	-1.857 (2.973)	-1.080 (2.212)	-1.302 (2.203)	2.376 (2.937)	3.293 (3.395)	2.434 (3.051)	2.142 (3.169)
Country-level variance	0.135*** (0.0388)	0.131** (0.0402)	0.129*** (0.0331)	0.140*** (0.0412)	0.887 (0.319)	0.778 (0.282)	0.911 (0.634)	0.827 (0.295)
Region-level variance	0.0381** (0.0127)	0.0381** (0.0127)	0.0378** (0.0126)	0.0376** (0.0124)	0.172*** (0.0417)	0.173*** (0.0418)	0.172*** (0.0418)	0.169*** (0.0418)
N	71868	71868	71868	71868	71454	71454	71454	71454

Standard errors in parentheses. $^{+}$ $P < .1$, * $P < .05$, ** $P < .01$, *** $P < .001$.

Table S8. Descriptive statistics, supplementary analyses

Descriptive Statistics, Supplementary Analyses			
	**OOP**	**GPs**	**Access Regulation Index (2016)**
Austria	18.1	0.77	0
Belgium	17.9	1.12	2
Czech Republic	14.2	0.70	1
Denmark	13.7	0.72	3
Estonia	22.7	0.70	3
Finland	19.4	1.26	3
France	6.9	1.54	2
Germany	12.9	0.67	1
Hungary	28.7	0.76	2
Ireland	15.1	0.74	3
Italy	22.8	0.74	3
Lithuania	32.5	0.88	3
Netherlands	11.9	0.80	2
Poland	23.4	0.22	3
Portugal	27.3	0.60	2
Slovenia	12.6	0.53	3
Spain	24.1	0.75	3
Sweden	15.4	0.65	2
Switzerland	27.8	1.13	2
United Kingdom	14.8	0.80	2
